# Impact of robotic assistance on the learning curve in endovascular interventions: exploring the role of operator experience with the CorPath GRX system

**DOI:** 10.1186/s42155-025-00529-y

**Published:** 2025-03-03

**Authors:** Tatjana Dell, Marilia B. Voigt, Alexander Isaak, Alexander Boehner, Claus Pieper, Narine Mesropyan, Patrick Kupczyk, Julian Luetkens, Daniel Kuetting

**Affiliations:** Department of Diagnostic and Interventional Radiology and Quantitative Imaging Lab Bonn (Qilab), University Hospital Bonn, Venusberg-Campus 1, Bonn, 53127 Germany

**Keywords:** Robotics, Endovascular, Learning curve

## Abstract

**Purpose:**

This study investigates how endovascular interventionalists adapt to using a robotic platform, specifically the CorPath GRX Robotic System, and examines the influence of prior manual catheterization experience.

**Materials and methods:**

An in-vitro trial was conducted to assess the adaptation to robot-assisted catheter-guided interventions in comparison to manual catheterization using a neurovascular phantom. Three interventional radiologists (beginner: no manual experience; intermediate: 3 years; expert: 10 years) with varying experience levels performed three corresponding robotic and manual guided superselective catherization tasks with three attempts per position with both approaches. Procedure time and total radiation dose was recorded.

**Results:**

For the beginner reduced intervention times were noted using the robot-assisted approach (mean: 123 s) in comparison to manual catheterization (mean: 257 s; *p* = 0.008), whilst no differences were seen in between procedure durations for the intermediate (mean: 71 s for manual versus 102 s for robotic; *p* = 0.388) and expert interventionalist (mean: 30 s for manual versus 42 s for robotic; *p* = 0.479). The beginner also benefited significantly from robot-assisted procedures with lower emitted total radiation doses (mean: 0.007 Gy for manual versus 0.003 Gy for robotic; *p* = 0.007), while no significant differences are observed for intermediate (mean: 0.002 Gy for manual versus 0.003 Gy for robotic; *p* = 0.137) and expert practitioners (mean: 0.0008 Gy for manual versus 0.001 Gy for robotic; *p* = 0.459).

**Conclusion:**

Robot-assisted platforms accelerate skill acquisition for beginners while maintaining efficiency for experienced practitioners. However, addressing costs and training requirements is essential for wider adoption and optimized outcomes.

## Background

It is widely acknowledged that the success, efficiency, and safety of endovascular procedures are closely tied to the expertise of the interventionalist. This expertise not only impacts procedural outcomes and duration but also influences critical factors such as radiation exposure to both patients and operators [1–3]. However, the introduction of robotic platforms for endovascular interventions presents a significant shift in procedural dynamics, raising questions about the transferability of traditional catheterization skills to this new technology. While robotic systems offer potential benefits, including enhanced precision and reduced radiation exposure for operators, it remains unclear whether the expertise developed for manual procedures seamlessly translates to robotic-guided interventions. This uncertainty poses a potential barrier to the integration of robotic platforms into clinical practice, emphasizing the need to investigate learning curves and skill adaptation for this emerging technology.

In this study, we aim to investigate how interventionalists with different levels of experience in manual catheter-guided procedures adapt to using a robotic platform (CorPath GRX Robotic System; Corindus Inc., Waltham, Massachusetts).

## Materials and methods

### Study design

**Fig. 1:**
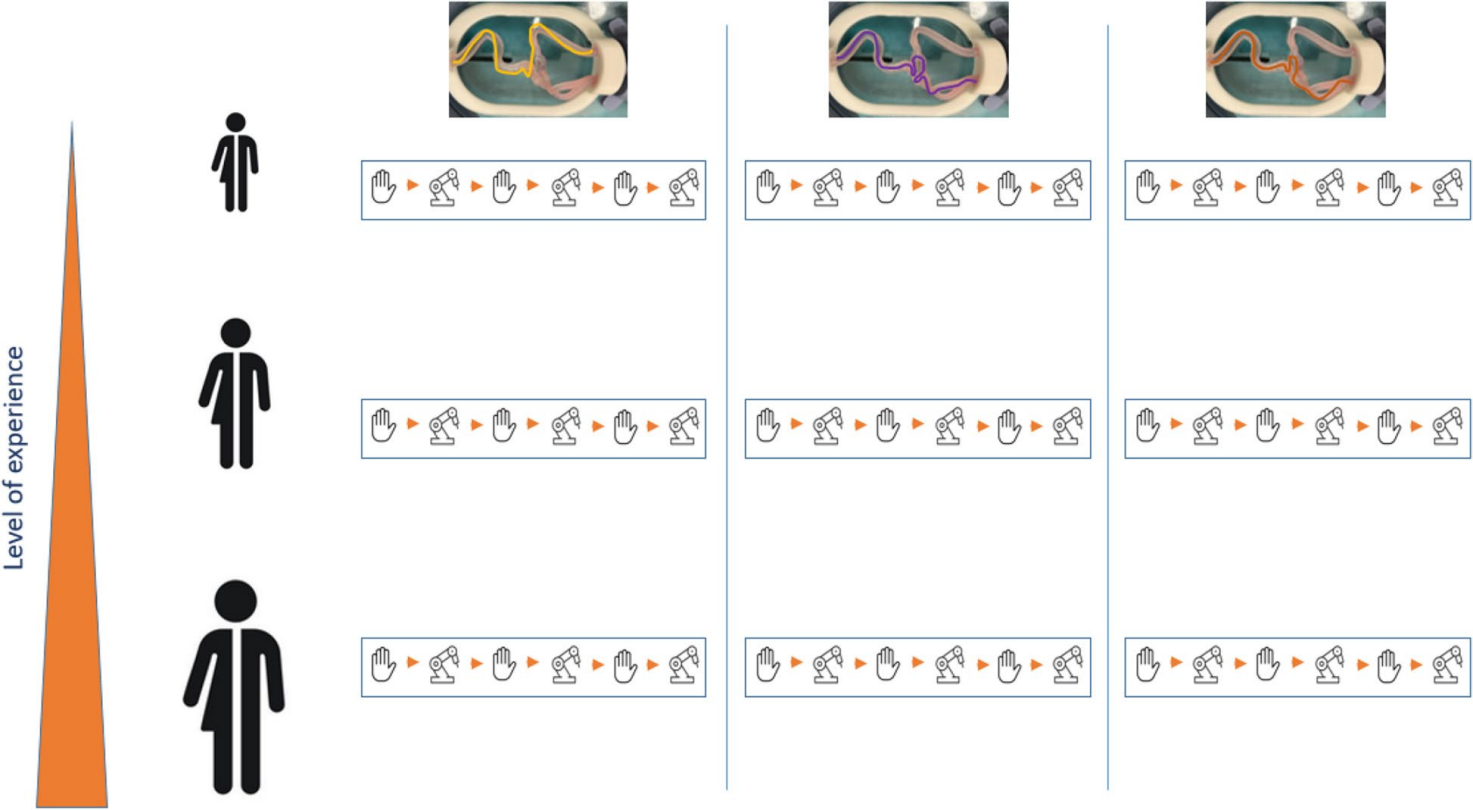
Study design. Three interventional radiologists with varying levels of experience (Beginner, Intermediate, Expert) alternated between freehand and robotic-assisted approaches. Each interventionalist performed six attempts per task position, which are colour-coded in the phantom images

This study is an in-vitro trial comparing robot-assisted and manually navigated catheter guided interventions. Three interventional radiologists with varying levels of experience in manually navigated catheter guided interventions (beginner: no prior experience in manual guided catheterization; intermediate: 3 years of experience; expert: 10 years of experience) and no experience in robotic guided interventions were tasked with probing three specific positions within a neurovascular phantom (Fig. 1). The procedures were alternated between freehand and robot-assisted approaches, with each interventionalist performing six attempts per position. The metrics recorded included procedure time (in seconds) and total radiation dose applied during an attempt (in Gy).

**Fig. 2:**
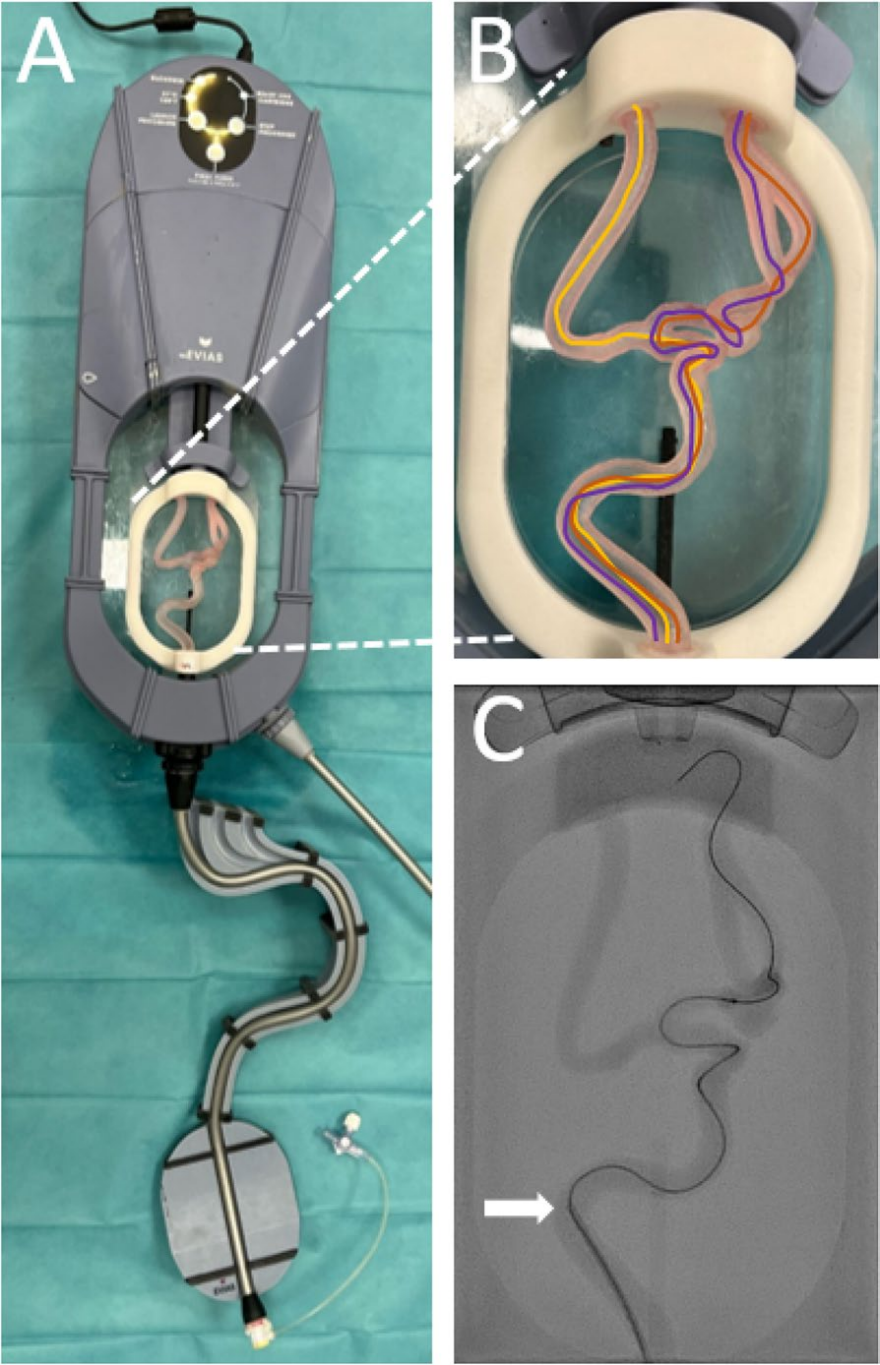
**A** Neurovascular model, EVIAS (Biomodex, France), used as the phantom in the study. **B** Three task positions, highlighted in different colors for identification. **C** An angiographic example image for one of the three positions. Arrow: Distal end of the macro catheter, defined as the starting point of the procedure

To ensure comparability between the parallel groups, the starting position and materials used were standardised. A 5F guide catheter was inserted into the phantom sheath with its straight tip positioned at the first curve of the flow model (Fig. 2), providing stable guidance during angiography. Through this guide catheter, a microcatheter (Renagade; Boston Scientific) was advanced to its distal tip with the aid of a microwire (Synchro ^2^®; Stryker). This distal tip served as the defined starting point for the procedure. The procedure was completed by positioning the microcatheter tip at the distal end of the flow model. The microwire was checked for functional integrity at the beginning of every attempt. In case integrity was no longer given, the wire was exchanged.

### Phantom model

The phantom used was a neurovascular simulation model, EVIAS (Biomodex, France; Fig. 2). A glycerol-water mixture was employed as a specialized fluid (BIOMODEX® BLOODSIM) to simulate the viscosity and flow characteristics of blood.

### Robotic model

**Fig. 3:**
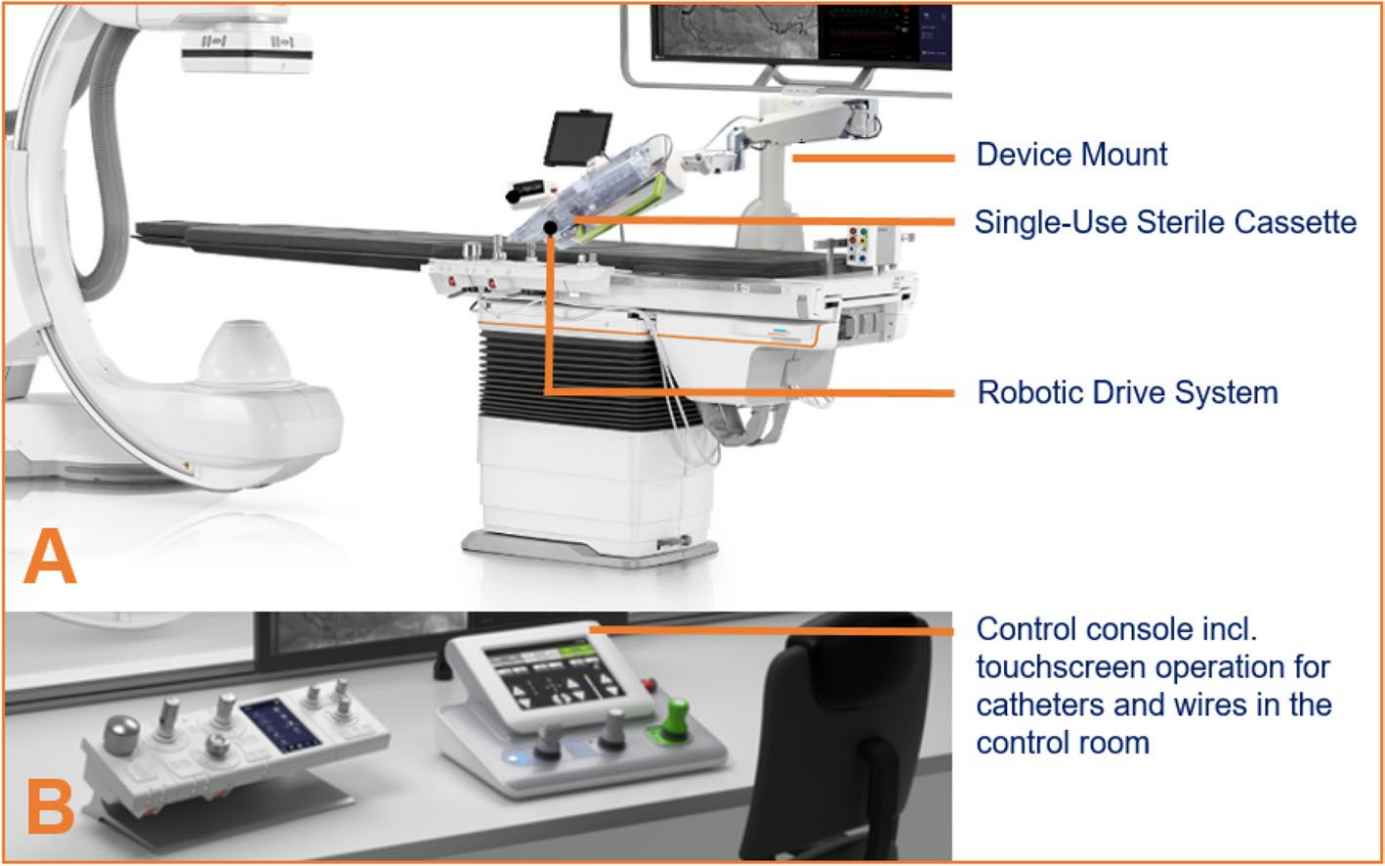
Representative procedure set-up using the CorPath GRX Robotic system. Bedside unit consisting of an articulating arm serving as the device mount, the robotic drive system and a single-use sterile cassette (**A**). Remote radiation-shielded workstation with monitors and controls, the ‘‘cockpit’’ (**B**)

A CorPath GRX Neurovascular Robotic System (Corindus, Waltham, MA) was utilized in this study. The key components, illustrated in Fig. 3, have been previously detailed in the literature [4–6]. In summary, the system comprises two main parts: a bedside unit and a robotic control console.

### Statistic analysis

Statistical analysis was performed using SPSS 29 (IBM, Armonk, New York, USA) with paired t-tests to assess intra-individual learning curves and descreptive analysis.

## Results

**Fig. 4:**
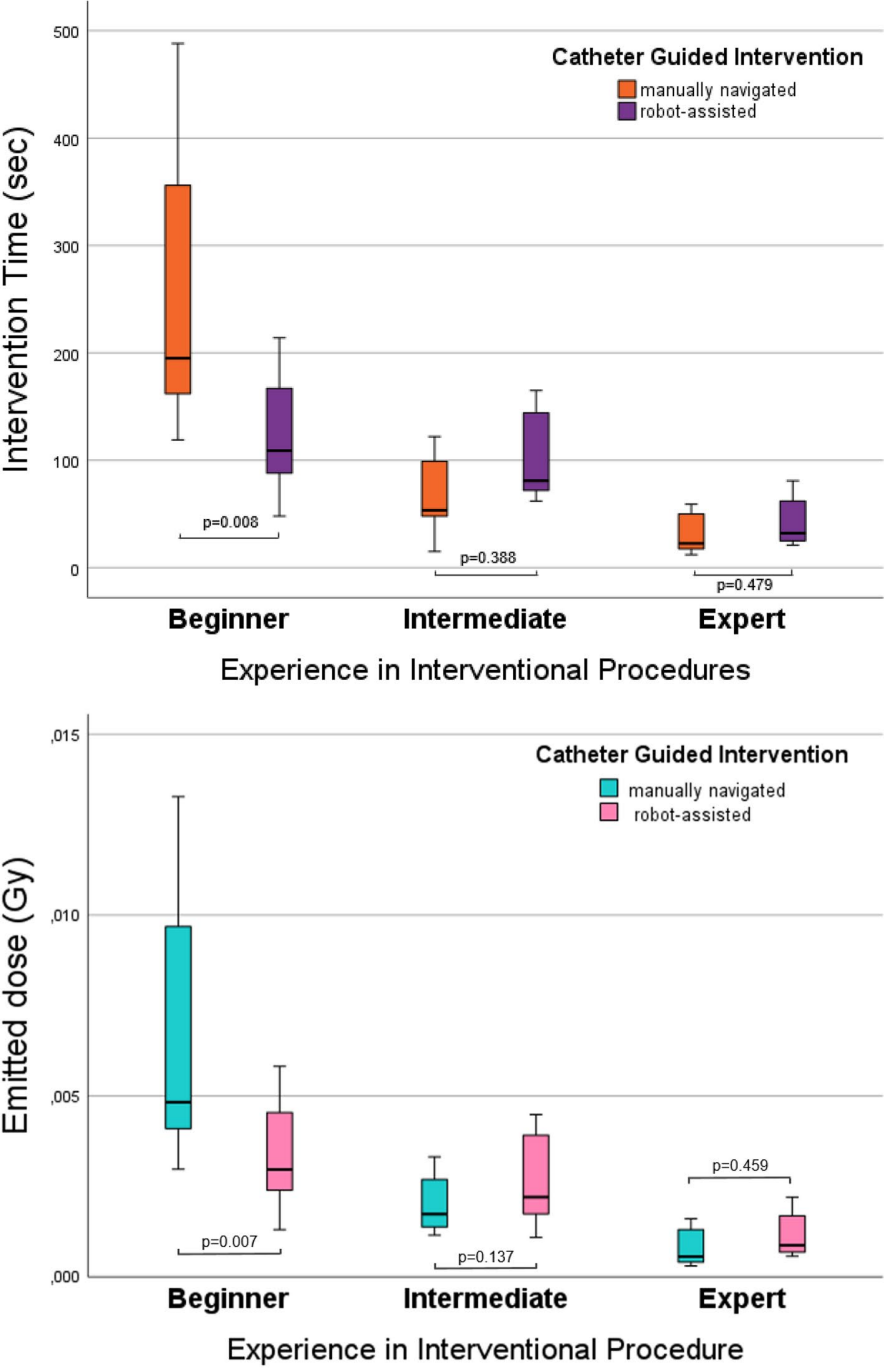
Comparison of intervention times (**A**) and emitted radiation dose (Gy; **B**) during catheter-guided interventions based on operator experience and navigation method

**Fig. 5:**
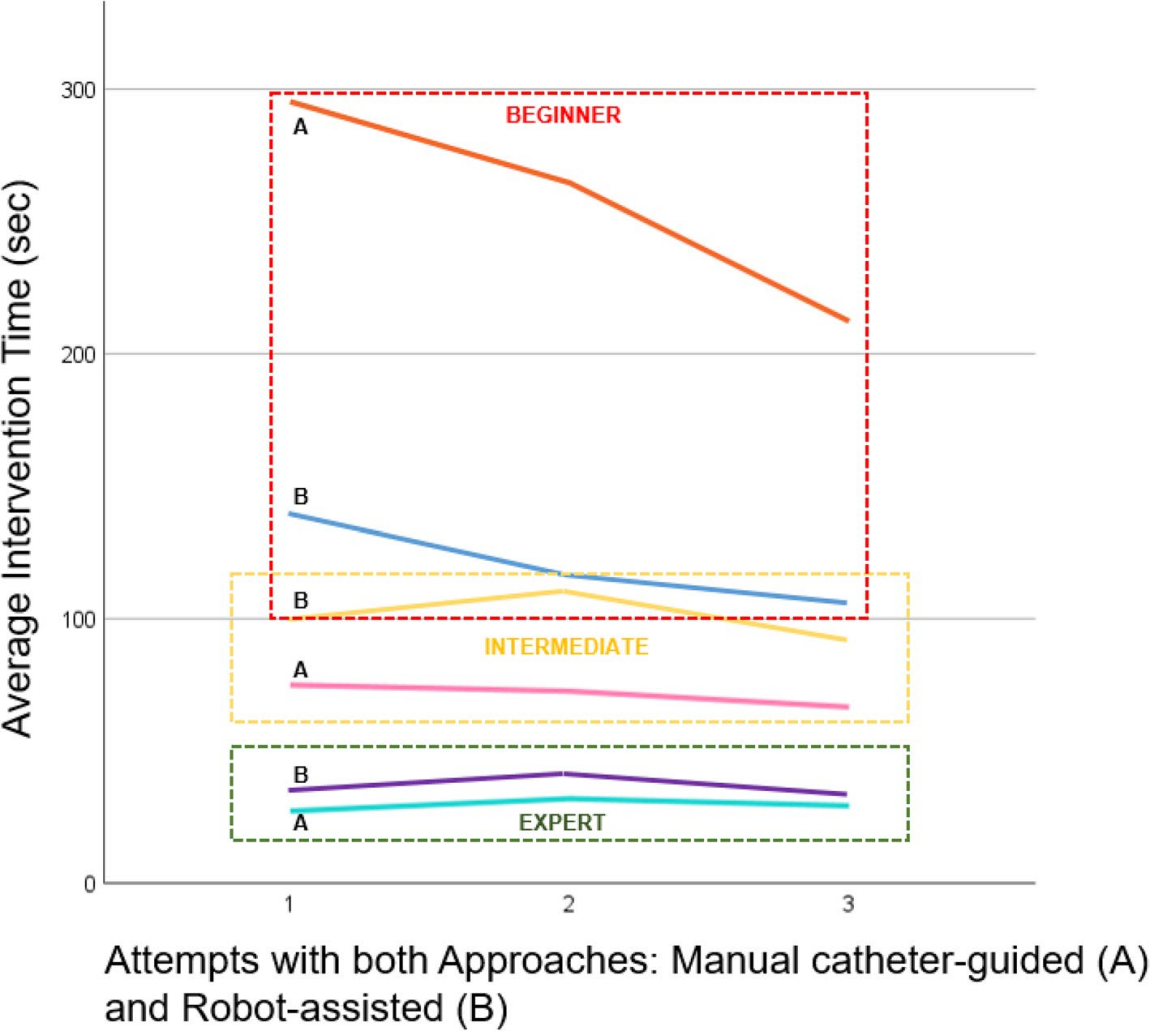
Learning curves for manual (**A**) and robot-assisted (**B**) catheter-guided procedures across experience levels (Beginner, Intermediate, Expert)

Figure 4 depicts the intervention times for three interventionalists (Beginner, Intermediate, Expert) performing catheter-guided procedures either manually as well as with robot assistance. For the beginner, robot-assisted procedures significantly reduced the intervention time (mean: 123 s for manual versus 257 s for robotic; *p* = 0.008), while no significant difference was observed for the interventionalist with intermediate experience (mean: 71 s for manual versus 102 s for robotic; *p* = 0.388) or the expert (mean: 30 s for manual versus 42 s for robotic; *p* = 0.479; *p* = 0.479). This is further visualized in the form of learning curves in Fig. 5. The average intervention time decreases with the number of attempts across all interventionalists, with the most significant improvement observed for the beginner. The expert consistently achieved high performance with minimal differences between the two methods. The interventionalist with intermediate experience showed moderate improvement, with comparable times between the approaches.

The results for total radiation dose show a similar trend: The beginner benefited significantly from robot-assisted procedures with lower emitted doses (mean: 0.007 Gy for manual versus 0.003 Gy for robotic; *p* = 0.007), while no significant differences are observed for the interventionalist with intermediate experience (mean: 0.002 Gy for manual versus 0.003 Gy for robotic; *p* = 0.137) and the expert (mean: 0.0008 Gy for manual versus 0.001 Gy for robotic; *p* = 0.459).

## Discussion

The use of robotic systems in clinical practice is firmly established across various medical disciplines. A prominent example is the da Vinci Surgical System, developed by Intuitive Surgical. Since its FDA approval in 2000, more than 12 million procedures have been performed globally using this system (https://isrg.intuitive.com/news-releases/news-release-details/intuitive-reaches-10-million-procedures-performed-using-da-vinci/). The da Vinci system has demonstrated superior outcomes, including reduced blood loss, shorter hospital stays, and improved postoperative recovery compared to open and laparoscopic operative techniques [7, 8].

Recent developments in robotic systems demonstrate promising utility also in endovascular procedures, offering great potential across various applications [4, 9]. An FDA-approved example is the CorPath GRX by Corindus Vascular Robotics, initially designed for percutaneous coronary interventions (PCI) and later adapted for peripheral vascular and neurovascular procedures. This system allows for precise control of catheters, microcatheters and wires, enabling operators to work remotely from outside the procedure room, thereby also reducing radiation exposure [10, 11].

However, introducing new techniques also comes with challenges. Little is known how interventionalists will adapt to robotic systems, whether expertise in manual catheterization is easily transferable and how the learning process on robotic systems differs to the conventional technique.

For the beginner, robotic assistance resulted in faster procedure completion compared to the traditional manual technique. This improvement is likely attributable to the intuitive design of the robotic system, which employs familiar interfaces from gaming such as joysticks and levers rather than wires and catheters used in manual procedures. Furthermore, the beginner exhibited a steeper learning curve using the robotic device, indicating that robotic platforms have the potential to expedite the training process for new interventionalists. Moreover, the total radiation dose for the beginner was significantly reduced when using the robotic system. This is particularly important, as beginners typically face longer intervention times during manual procedures, which increases their cumulative exposure to radiation.

In contrast, the intermediate practitioner demonstrated comparable performance across both techniques, with procedure times and total radiation dose remaining similar for robotic and manual approaches. This suggests that while the semi-expert is adept at manual techniques, their transition to robotic systems is seamless.

For the expert, manual techniques were consistently faster. The extensive experience with traditional methods allowed him to complete procedures more efficiently than with robotic assistance. The robotic system inherently limits speed, allowing experts to perform certain tasks, such as moving micro-catheters and wires, more quickly than the robot can. Nevertheless, the fundamental knowledge and skills from manual techniques remain applicable to robotic systems, allowing experts to adapt more swiftly than intermediate practitioners, who, in turn, learn faster than beginners. This suggests that familiarity with manual procedures provides a significant advantage in adapting to robotic-assisted interventions.

Despite the theoretical advantages robotic systems offer, the use of currently available robotic platforms is linked with several issues that hinder their widespread adoption in routine clinical practice. A major downside is the extended setup time, particularly for novice users, which decreases efficiency compared to traditional methods. Moreover, the high initial investment and ongoing maintenance costs present substantial financial barriers, particularly as there are currently no reimbursement models or compensation frameworks specifically tailored to the use of robotic platforms for endovascular procedures. These costs must be carefully balanced against the potential benefits, such as reduced operator fatigue, enhanced procedural precision, and decreased radiation exposure.

While robotic platforms have become integral to certain surgical specialties, such as urological and visceral procedures—including prostatectomies and rectal cancer surgeries—these fields also encounter challenges. These include high operational costs, the requirement for specialized training programs, and the limited tactile feedback inherent to robotic systems. However, the reduced invasiveness and improved outcomes associated with the use of robotic platforms have outweighed these limitations. Whether this balance holds true for the use of robotic platforms in endovascular procedures remains to be established.

One of the key aspects for successful implementation of robotic-assisted interventions is the efficient transfer of previously acquired manual skills to robotic techniques. While our findings indicate that beginners benefit from a steep learning curve, experienced operators may require adapted training approaches to optimize efficiency when transitioning to robotic platforms. Future studies should focus on structured training programs that facilitate skill transfer and evaluate strategies such as simulation-based learning or haptic feedback systems to bridge the gap between manual and robotic procedures.

## Conclusion

Robotic platforms simplify interventional acquisition for beginners and maintain efficiency for experts, with benefits like reduced procedure times and total radiation dose for novices. However, challenges such as high costs and extended setup times hinder widespread use. Despite these barriers, robotic systems show promise in enhancing precision and safety, suggesting potential for broader adoption with advancements in training and cost management.

## Data Availability

The datasets analysed during the current study are available from the corresponding author on reasonable request.
